# F156. LONGITUDINAL WORKING MEMORY FUNCTIONAL DYSCONNECTIVITY REFLECTS HETEROGENEITY IN INDIVIDUALS AT ULTRA HIGH RISK FOR PSYCHOSIS

**DOI:** 10.1093/schbul/sby017.687

**Published:** 2018-04-01

**Authors:** Siwei Liu, Jimmy Lee, Jesisca Tandi, Chenhao Wang, Joseph K W Lim, Newfei Ho, Joann Poh, R Alison Adcock, Richard Keefe, Stephen Wood, Ranga Krishnan, Michael Chee, Juan Zhou

**Affiliations:** 1 Duke-NUS Medical School; 2 Institute of Mental Health, Singapore; 3 Centre for Cognitive Neuroscience, Duke-NUS Medical School; 4 Duke University; 5 University of Birmingham

## Abstract

**Background:**

Variation in trajectories of Ultra high-risk (UHR) psychosis mental state posts challenge to schizophrenia prevention or onset delay intervention. Our previous work described the heterogeneity at this prodromal stage of schizophrenia in both brain structure changes and resting-state functional network differences. Functional dysconnectivity can be one of the altered brain substrates underlying clinical symptoms. Lower resting-state functional connectivity (FC) within the salience network (SN) in schizophrenia was detectable at the UHR stage. FC between the frontoparietal network (FPN) and the SN was disrupted when network integration fell apart in the UHR state.

FPN and SN are important for working memory (WM), which is largely compromised in schizophrenia and lesser in UHR group. Our previous work showed that WM task-related activation in the FPN and SN differed between the UHR and controls. Importantly, such differences varied with WM demands. Evidence has demonstrated that compared to resting-state FC, task-based FC may better predict behavioral performance. However, the WM-related FC in UHR group and its longitudinal changes are still largely unknown.

To cover the gap, we sought to examine the heterogeneity in the WM task-related FC changes in a group of UHR participants over time. We expected WM-related FC would link to individual differences in clinical trajectories.

**Methods:**

Based on the longitudinal changes of UHR state within 2 years, participants were divided into 3 groups: 42 controls, 34 UHR remitters (UHR-R) and 42 UHR non-remitters (UHR-NR). We acquired fMRI (TR/TE = 2000/30 ms, 3 x 3 x 4 mm3, 28 slices) when participants performed WM task at different WM demands, varying from information maintenance alone (low) to requiring both maintenance and manipulation (high). We used seed-based approach (gPPI) to compare task-related FC of the FPN and the SN among groups. Voxel-wise FC with six seeds (bilateral anterior insula, parietal, and dorsal lateral prefrontal cortex, identified based on task activation) was regressed on WM demands and groups, controlling for age, gender, education, ethnicity, handedness and task accuracy. Linear mixed modeling methods were used to test longitudinal FC changes and the association between FC and clinical syndromes.

**Results:**

Task performance was worse at high WM demand as expected, but no significant difference was found between groups or over time. Compared to controls, higher FC between the FPN (superior parietal gyrus) and the SN (insula) at low demand was observed in the UHR-NR group at baseline. Within the SN, WM-demand related FC between right insula and thalamus varied among 3 groups: low FC at low demand and high FC at high demand in controls; high FC at low demand but low FC at high demand in the UHR-R group. In contrast, UHR-NR group had high thalamus-insula FC in both WM demands.

Moreover, longitudinal FC increase only occurred within the SN at high WM demand in the UHR-R group, while other task-related baseline group differences of FC remained stable over time. Importantly, the rate of intra-SN increase of FC over time at higher WM demand was associated with decline of the positive psychosis syndromes in the UHR-R group.

**Discussion:**

In support of the functional dysconnectivity hypothesis, our study indicated that UHR state was accompanied by altered brain FC during WM task performance. In contrast to lower SN FC at rest, UHR state showed SN hyper-connectivity in task with low WM demand, suggesting the importance of studying UHR both at resting and in task. Importantly, intra-SN FC increase at high WM demand was linked to positive psychosis syndrome reduction in remitters, while no FC changes if UHR state persisted. We argued that task FC could reflect the clinical heterogeneity of the UHR group.

